# Supplementary material for: Science development study for the Atacama Large Aperture Submillimeter Telescope (AtLAST): Solar and stellar observations
Source: Open Res Eur. 2024 Dec 12;4:140. Originally published 2024 Jul 9. [Version 2] doi: 10.12688/openreseurope.17453.2 (PMC11320049; doi:10.12688/openreseurope.17453.2)
Supplement: Peer review history

# Reviewer report: Reviewer response for version 2

**DOI:** 10.21956/openreseurope.20507.r48569

**Published:** 2025-02-07

**Authors:** Alexander G.M. Pietrow

**Affiliations:** 1 Leibniz Institute for Astrophysics Potsdam, Potsdam, Germany

Thank you for the prompt and thorough revision of the article. I believe its readability for non-experts has significantly improved, and the citations are now well integrated. All of my previous concerns have been addressed to my satisfaction. Based on these revisions, I would like to change the status of my review to ‘Approved’.

Is the case presented with sufficient detail to be useful for teaching or other practitioners?

Partly

Is the work clearly and accurately presented and does it cite the current literature?

Partly

If applicable, is the statistical analysis and its interpretation appropriate?

Not applicable

Are all the source data underlying the results available to ensure full reproducibility?

Not applicable

Are the conclusions drawn adequately supported by the results?

Yes

Is the background of the case’s history and progression described in sufficient detail?

Yes

Reviewer Expertise:

Solar physics, Sun-as-a-star observations, flares, and spectropolarimetric inversions.

I confirm that I have read this submission and believe that I have an appropriate level of expertise to confirm that it is of an acceptable scientific standard.

# Reviewer report: Reviewer response for version 2

**DOI:** 10.21956/openreseurope.20507.r48570

**Published:** 2024-12-26

**Authors:** Malcolm Druett

**Affiliations:** 1 The University of Sheffield Department of Automatic Control and Systems Engineering, Sheffield, England, UK

Thank you for the invitation to review the revised version of the article. I believe that "Approved" status for this article is warranted based on the updates to the manuscript.

Is the case presented with sufficient detail to be useful for teaching or other practitioners?

Partly

Is the work clearly and accurately presented and does it cite the current literature?

Partly

If applicable, is the statistical analysis and its interpretation appropriate?

Not applicable

Are all the source data underlying the results available to ensure full reproducibility?

Not applicable

Are the conclusions drawn adequately supported by the results?

Yes

Is the background of the case’s history and progression described in sufficient detail?

Yes

Reviewer Expertise:

solar flares, sun: chromosphere, radiative transfer, magnetohydrodynamics, sun as a star

I confirm that I have read this submission and believe that I have an appropriate level of expertise to confirm that it is of an acceptable scientific standard.

# Reviewer report: Reviewer response for version 1

**DOI:** 10.21956/openreseurope.18862.r42515

**Published:** 2024-08-12

**Authors:** Malcolm Druett

**Affiliations:** 1 The University of Sheffield Department of Automatic Control and Systems Engineering, Sheffield, England, UK

**Review Summary**

 I was very pleased to read this paper, as I consider the instrument under to discussion to be a very exciting addition to the fleet of instruments we can use to study the Sun and other stars. This paper highlights several promises to advance our understanding of many active research themes using the instrument: coronal heating, the nature of prominences, the production of CMEs and long-term activity cycles. Moreover, this instrument has the potential to add to the joining up of science with millimeter wavelength range co-observations, as well as strengthening our understanding of the links, differences, and gradients in behaviours between our Sun and other stars.

 One point I would appreciate the authors addressing is that I was expecting to be first presented with clear, concise summary of the proposed observational parameters before the science use cases in order to be able to understand and evaluate these. The ordering of the sections is such that if you are unfamiliar with the instrument, as many readers may be, you must simply accept the claims about the suitability for each science use case when reading through the paper in order. This concern could be addressed through clear referencing to specific points in later sections, a summary table, or restructuring of the paper.

 Also in some of the sections where science use cases are presented, I recommend stronger links to be drawn between the observational capabilities of this instrument and the constraints required to address those science questions. Without these links made explicit to the reader I believe that the current version of the manuscript has sections that are diluted in informational value, with un-cited, uncorroborated, or decontextualised claims.

 The author list boasts an exceptional array of talent that I am certain is capable of addressing these concerns. I recommend edits are made before acceptance.

**Introduction**

 (1) "The thermal and magnetic structure of the chromosphere is complex and highly dynamic with a plethora of physical processes at work, many of which induce notable deviations from equilibrium conditions (for example in the ionisation state of hydrogen). The largest features like filaments can span substantial fractions of the solar diameter (∼30’)"

 This phrasing suggest filaments to be chromospheric plasma rather than cool dense coronal plasma. I recommend the authors to re-phrase this.

 (2) "and thus contributing to the solution of many open questions (see Wedemeyer et al., 2016, and references therein)."

 This sentence appears to be vague. Perhaps a few examples of key science questions addressed would be instructive to the reader.

 (3) The following point is relating to the discussion in this section, I would like to hear the author's feedback on this, but it is not necessarily something that needs to be included in the text unless there are elements that the authors would deem worthwhile enough for inclusion, for example to bolster the strength of claims made within the later sections.

 Many of the current authors were also involved in the publications of Chintzoglou et al. 2021 I & II [references 1,2]. Could I ask the authors comment on the findings stated in the summary and conclusions of Chintzoglou et al. 2021 II, regarding the discrepancy between the formation heights of Band 6 found in this study and earlier ones.

 Do you believe that discussion of formation height for different solar features have already been resolved clearly to the satisfaction of the community? If not what are the knock-on error bars like for the key science cases based on these uncertainties or disagreements?

 (4) "Despite already large success of ALMA in advancing solar physics (e.g. Brajša et al., 2018; Heinzel et al., 2022; Labrosse et al., 2022; Menezes et al., 2022; Oliveira e Silva et al., 2022; Rodger et al., 2019; Selhorst et al., 2019; Shimojo et al., 2017a; Skokić et al., 2023; Valle Silva et al., 2021), there are important science cases that require different observational capabilities"

 I recommend that you split these advances into a few themes and at least mention what themes these are that ALMA has advanced. It's the second claim in the introduction of big contributions to advancements in the field with no specific examples yet provided to the reader. Further interesting advances from authors not associated with this work also perhaps warrant consideration in such a list. for example Multiple papers by Da Silva Santos, Chinzoglou, Molnar spring to mind, but there are also others [references 1-8]. I am not requesting an in-depth discussion or an exhaustive list, but some context for the reader who is not familiar with these works.

**Key Science Cases**

 (5) "The chromosphere is an integral part of the solar atmosphere and as such plays an important role in the transport of energy and matter. These are essential for understanding the heating of the corona, the origins of the solar wind, and the drivers of solar activity and space weather. There is a plethora of physical processes involved, which are entangled in complicated dynamic ways, rendering the understanding of the workings of our Sun a challenging undertaking."

 (a) citations needed.

 (b) "The chromosphere is an integral part of the solar atmosphere and as such plays an important role in the transport of energy and matter."

 This sentence currently has low informational value. I suggest either

 - simply stating that the chromosphere plays an important role in the transport of energy and matter with some citations

 or

 - actually explaining what you mean by a little in terms of it being "an integral part" of the solar atmosphere and why. For example, one could argue this in terms of the behaviour changes either side of the plasma beta=1 layer, wave propagation, and non-equilibrium conditions. I am not a fan of characterising something as complicated or shrouded in mystery for the reader to understand that there are unresolved, interesting, and valuable science questions. It would be great if the authors could add some detail to pull back the curtain of mystery and complexity a little.

 (6) "As detailed in the sections below, AtLAST (Mroczkowski et al., 2024) opens up a new window to science cases that cannot be addressed with existing sub-mm telescopes. In addition, some solar science cases for ALMA (see Bastian et al., 2018; Wedemeyer et al., 2016, and references therein; see also Figure 1) and LLAMA (Lépine et al., 2021) are potential solar use cases for AtLAST, too, thus creating synergies between these observatories. These science cases concern the thermal structure of the solar atmosphere including the thermal structure of prominences and filaments, the related transport of energy and mass, and phenomena related to solar activity. In the following, the key science cases for AtLAST are highlighted."

 (a) I do not see the authors linking Figure 1 to anything stated above. The images show two time points in the solar cycle. To clarify, I am certain that solar cycle data from ALMA has valuable information. However, since none is mentioned or explained here, figure 1 appears to be only a visually satisfying image. Please provide some context in the caption or the text. This could even be a reference to the later point in the paper where this figure is discussed.

 (b) Are you saying these listed science cases will be addressed in the following sections? If so, then I request you say that. If not then the statement is too brief to provide any information of value to the reader and I recommend adding citations, context, or both.

**2.1 Thermal structure and the atmospheric heating problem**

 (7) The first part of this section is very nicely stated, although again lacking any citations beyond one early paper relating to the coronal heating problem. Please add citations.

 "In principle, identifying these mechanisms and understanding their contributions requires quantitative and precise measurements that capture the thermal, magnetic, and kinetic state of chromospheric plasma over time and in three spatial dimensions. AtLAST has the potential to provide such data."

 At this point in the paper, the reader has not been provided with an overview of the capabilities of the instrument, nor have you provided citations with reasoned explanations or estimated of what observational capabilities could be required to derive these properties at the cadences and spectral/spatial resolutions required. Thus, unless the reader is already previously aware of all these details they have to accept the claim on trust. As stated in the summary I recommend this concern could be adequately addressed with context, references to later sections, or re-structuring of the paper.

**2.2 Solar Flares**

 (8) "AtLAST is poised to make significant contributions in addressing numerous unresolved inquiries pertaining to solar flares, a prominent conundrum within contemporary solar physics that continues to be a highly dynamic area of investigation. Although numerous intricacies remain shrouded in mystery, it is evident that solar flares manifest as a result of the dynamic reconfiguration and interconnection of magnetic fields within the solar atmosphere."

 Citations needed here. But I thank the authors of this section for providing sufficient citations elsewhere.

 (9) "And yet, it cannot be ruled out that even stronger flares exceeding the Carrington event of 1859 could occur with disastrous consequences for our technology-dependent modern society. Such so-called super-flares are observed for other (solar-like) stars (Maehara et al., 2012) (see Section 2.5). The study of strong solar flares, to which AtLAST can contribute in new ways, is thus of utmost importance."

 At this point in the paper without any solid evidence provided of how AtLAST can contribute, we are taking this "utmost importance" on faith. I recommend the authors to talk about the ways in which it can contribute explicitly. For example describing the observational capabilities, cadences, resolutions, atmospheric parameters, and how these relate to the flare science questions identified.

 (10) Figure 2:

 I assume the close association of mm-continuum radiation with brightness temperature will providing information to answer particular science questions. The relationship in flares is nicely demonstrated by the figure. Please point this out, and mention some of the science cases this will address and explain how the information will help.

 (11) "Observations of such sub-mm flares might also provide key information about the formation of hot onsets in solar flares (da Silva et al., 2023; Hudson et al., 2021)."

 What sort of key information? Why "might"? Can you analyse whether the information obtained would help address the science questions? I recommend talking a bit about the science questions, the observational requirements and how AtLAST can provide observations that meet those requirements.

 (12) "Addressing these aspects with AtLAST would require high spectral, spatial and temporal resolution and preferably full polarisation"

 And can/do we have that? The observational capabilities of the instrumentation have not been summarised or discussed. Please address whether you believe this is feasible and justify your position to the reader.

**Solar Prominences**

 (13) "These prominences are among the longest-lasting solar phenomena with lifetimes of several days to weeks. The large field of view of AtLAST, combined with its good spatial resolution and the ability to observe the Sun on daily bases for extended periods, will offer us opportunities to study the questions of the prominence origin, evolution, and eventual instability."

 The paragraph below this one makes a nice case for mm studies of prominences.

 I recommend that the discussion could be supplemented in several ways:

 (a) citations where appropriate.

 (b) are there physical processes relating to prominence formation/evolution/instability that occur at scales and cadences you anticipate to be evident at AtLAST's resolution (please also state that resolution), which are not currently accessible via existing solar mm instrumentation?

 (c) it may also be worth mentioning the validation and calibration you could perform on estimates of plasma quantities that are otherwise made using inferences from complicated optically thick radiative transfer.

 (14) "because the drivers that make the prominence magnetic field configurations unstable and erupt are still not well understood." & "However, it is unclear exactly what sets off these eruptions."

 Repetition

**Solar Activity Cycle**

 (15) I recommend that this section could do with a few more citations for the results you discuss - if possible not so dominantly from one of your authors, Menezes. Also please define TP in the text.

 (16) "Please note that the Sun’s rotation depends on latitude with a period of down to 24 days at the equator (see, e.g., Schou et al., 1998; Snodgrass, 1983). Any long-lived feature like an Active Region would thus move across the visible disk of the Sun in less than two weeks. However, at the resolution anticipated for AtLAST (see Figure 3), features like Active Regions and the sunspots (Solanki, 2003) therein would evolve significantly on a daily timescale. **This use case** would therefore be addressed best via full-disk mapping of the Sun at multiple frequency bands, once per day."

 Please clarify which use case you are referring to - Are you talking about studying the solar cycle in general or some specific observational case within that?

 (17) "New insights regarding the Sun’s activity variations and thus the long-term evolution of our host star could be transferred to other stars. This Sun-as-a-star approach has recently received increasing attention in the context of next-generation exoplanet observations for which it is crucial to separate observable exoplanet signatures from the host star’s “background radiation”."

 Citations needed.

**The Solar Stellar Connection**

 (18) This section does several things very well that should be highlighted for interpreting my other comments in this review.

 (a) cites papers when claims regarding physical processes are made that relate to the science cases.

 (b) relates stated capabilities and resolutions/cadences of the instrument directly to the use cases under discussion.

 The spectrum of cited authors and findings within the field is rather limited, I suggest a more comprehensive coverage, but other than this the I have no notes on the section.

**3 Observing the Sun with AtLAST**

 (19) The information in this section would have been useful to know earlier, or at least be referenced from the earlier sections. Also given some of the abbreviations are defined in section 3 and used in section 2 I would assume there was already some debate regarding this ordering. Please make section 2 easier to follow for first time readers by linking the claims to information from section 3.

 (20) "The Sun evolves on short time scales which prohibits long integration/scan times."

 I recommend changing the word "prohibits" to another word, as this doesn't really prohibit long integration times, it just devalues them for resolving transient structures.

 (21) "While it has yet to be seen if spectral lines, mostly hydrogen recombination lines and potentially CO, can be detected at millimeter wavelengths, they would have large diagnostic potential for assessing the thermal, kinetic and magnetic state of the chromospheric plasma."

 Could you comment on whether there is any ongoing effort to look into the existence of these lines within the proposed spectrum? Do you intend to conduct such a search in the early use cases?

**3.1.3 Sensitivity, Integration time, Dynamic range**

 (22) Regarding the points made in the final paragraph, please could you comment on the outlook for being able to cover the necessary for studying flares? Perhaps some mention of this would be useful in the text too. I do believe there would be very useful information to glean about flare ribbons, condensation and evaporation even from a more limited dynamic range.

**3.1.4 Polarisation**

 (23) "magnetograms obtained from the spectral lines in the photosphere (i.e., the Sun’s surface layer)"

 This is the 4th instance of the word photosphere. If you wish to explain what it is, I would recommend doing so at the first instance.

 (24) "Reliably measuring the magnetic field in the solar chromosphere above, i.e. the layer that would be observed with AtLAST, is still a current technological challenge, again with much diagnostic potential."

 Citations to works from Pietrow, Morosin, and Vissers plus any the authors wish would be useful here [Refences 9-11].

 (25) "Early observations with ALMA at a frequency of 100 GHz, which were obtained as science verification data in Band 3 (ADS/JAO.ALMA#2011.0.00011.E),"

 I recommend considering if this is the best way to cite this data. Perhaps providing a footnote or a reference to its description elsewhere in the document that says what this is and how to obtain it.

**3.2 Observing modes**

**3.2.1**

 (26) "with respect to the single-dish Total Power scans with ALMA antennas"

 TP is now unabbreviated. I agree with the final statement of this section, that would be a most welcome addition to our field.

**3.3 Instrumentation**

 (27) "the resulting diameter of the FOV is approximated as d fov ≈ ..."

 For clarity, I recommend using a format that makes it explicitly clear what is, and what is not, under the large square root. This could also be achieved satisfactorily with parentheses.

**3.3.1**

 (28) "The details of the detector setup have to be investigated in detail. That includes the spacing between pixels, the total number of pixels, and the resulting instantaneously covered FOV. A fast small circular scanning pattern could be used to fill intermediate pointings. Combining data from circular scans (if possible on a second time scale or at least <10 s) would then allow to produce data with higher angular resolution. A detailed study is currently in preparation (Kirkaune et al. in prep.)."

 Have the matters been studied in detail, or are being studied in detail (in prep)? If the former, I would recommend an additional citation to a published work referenced in first part of this paragraph.

**3.3.2**

 (29) "Such a detector would allow for exploiting the spectral domain but comes at the price of a much smaller number of detector elements as compared to multi-chroic continuum cameras (see Section 3.3.1),"

 Why is this? Simply by re-assignment of those pixels?

 and a typo later: overal -> overall

**3.3.3**

 (30) "In any case, a realistic evaluation of the scientific potential of IFUs will require a dedicated study."

 Please comment on whether you have plans to perform that study.

Is the case presented with sufficient detail to be useful for teaching or other practitioners?

Partly

Is the work clearly and accurately presented and does it cite the current literature?

Partly

If applicable, is the statistical analysis and its interpretation appropriate?

Not applicable

Are all the source data underlying the results available to ensure full reproducibility?

Not applicable

Are the conclusions drawn adequately supported by the results?

Yes

Is the background of the case’s history and progression described in sufficient detail?

Yes

Reviewer Expertise:

solar flares, sun: chromosphere, radiative transfer, magnetohydrodynamics, sun as a star

I confirm that I have read this submission and believe that I have an appropriate level of expertise to confirm that it is of an acceptable scientific standard, however I have significant reservations, as outlined above.

## References

[1] : ALMA and IRIS Observations of the Solar Chromosphere. I. An On-disk Type II Spicule. *The Astrophysical Journal* .2021;906(2) : 10.3847/1538-4357/abc9b1 10.3847/1538-4357/abc9b1

[2] : ALMA and IRIS Observations of the Solar Chromosphere. II. Structure and Dynamics of Chromospheric Plages. *The Astrophysical Journal* .2021;906(2) : 10.3847/1538-4357/abc9b0 10.3847/1538-4357/abc9b0

[3] : The multi-thermal chromosphere. *Astronomy & Astrophysics* .2020;634: 10.1051/0004-6361/201937117 10.1051/0004-6361/201937117

[4] : ALMA observations of transient heating in a solar active region. *Astronomy & Astrophysics* .2020;643: 10.1051/0004-6361/202038755 10.1051/0004-6361/202038755

[5] : Heating of the solar chromosphere through current dissipation. *Astronomy & Astrophysics* .2022;661: 10.1051/0004-6361/202243191 10.1051/0004-6361/202243191

[6] : Subarcsecond Imaging of a Solar Active Region Filament With ALMA and IRIS. *Frontiers in Astronomy and Space Sciences* .2022;9: 10.3389/fspas.2022.898115 10.3389/fspas.2022.898115

[7] : Solar Chromospheric Temperature Diagnostics: A Joint ALMA-Hα Analysis. *The Astrophysical Journal* .2019;881(2) : 10.3847/1538-4357/ab2ba3 10.3847/1538-4357/ab2ba3

[8] : High-frequency Wave Power Observed in the Solar Chromosphere with IBIS and ALMA. *The Astrophysical Journal* .2021;920(2) : 10.3847/1538-4357/ac1515 10.3847/1538-4357/ac1515

[9] : Inference of the chromospheric magnetic field configuration of solar plage using the Ca II 8542 Å line. *Astronomy & Astrophysics* .2020;644: 10.1051/0004-6361/202038750 10.1051/0004-6361/202038750

[10] : Spatio-temporal analysis of chromospheric heating in a plage region. *Astronomy & Astrophysics* .2022;664: 10.1051/0004-6361/202243461 10.1051/0004-6361/202243461

[11] : Non-LTE inversions of a confined X2.2 flare. *Astronomy & Astrophysics* .2021;645: 10.1051/0004-6361/202038900 10.1051/0004-6361/202038900

# Response

**Published:** 2024-11-13

**Authors:** Sven Wedemeyer

We would like to thank the referee for the useful suggestions. We considered all comments, which resulted in an improved manuscript. Please note that the revised version takes into account the comments from all three referees. We added 81 additional references and updated Figs. 3 and 4. In the following, we address all comments in detail.  

 ========================================================================

 REFEREE> One point I would appreciate the authors addressing is that I was expecting to be first presented with clear, concise summary of the proposed observational parameters before the science use cases in order to be able to understand and evaluate these. The ordering of the sections is such that if you are unfamiliar with the instrument, as many readers may be, you must simply accept the claims about the suitability for each science use case when reading through the paper in order. This concern could be addressed through clear referencing to specific points in later sections, a summary table, or restructuring of the paper. 

 ANSWER> We thank the referee for this comment but would like to point out that the purpose of this manuscript is to do the opposite: Summarise key science cases that can be addressed with a large single-dish (sub)mm telescope and then derive instrumental requirements. However, we take this comment seriously and modified the manuscript in this direction.  

 ========================================================================

 REFEREE> Also in some of the sections where science use cases are presented, I recommend stronger links to be drawn between the observational capabilities of this instrument and the constraints required to address those science questions. Without these links made explicit to the reader I believe that the current version of the manuscript has sections that are diluted in informational value, with un-cited, uncorroborated, or decontextualised claims. 

 ANSWER> We would like to refer to our answer to the previous point and will make changes at the appropriate places. We also added two clarifying sentences at the end of the introduction.  ========================================================================

 (1) "The thermal and magnetic structure of the chromosphere is complex and highly dynamic with a plethora of physical processes at work, many of which induce notable deviations from equilibrium conditions (for example in the ionisation state of hydrogen). The largest features like filaments can span substantial fractions of the solar diameter (∼30’)"

 This phrasing suggest filaments to be chromospheric plasma rather than cool dense coronal plasma. I recommend the authors to re-phrase this.

 ANSWER> We modified this passage accordingly.  ========================================================================

 (2) "and thus contributing to the solution of many open questions (see Wedemeyer et al., 2016, and references therein)." This sentence appears to be vague. Perhaps a few examples of key science questions addressed would be instructive to the reader.

 ANSWER> We modified this passage accordingly.  

 ========================================================================

 (3) The following point is relating to the discussion in this section, I would like to hear the author's feedback on this, but it is not necessarily something that needs to be included in the text unless there are elements that the authors would deem worthwhile enough for inclusion, for example to bolster the strength of claims made within the later sections. Many of the current authors were also involved in the publications of Chintzoglou et al. 2021 I & II [references 1,2]. Could I ask the authors comment on the findings stated in the summary and conclusions of Chintzoglou et al. 2021 II, regarding the discrepancy between the formation heights of Band 6 found in this study and earlier ones.

 Do you believe that discussion of formation height for different solar features have already been resolved clearly to the satisfaction of the community? If not what are the knock-on error bars like for the key science cases based on these uncertainties or disagreements? 

 ANSWER> That is a very good question. One of the surprises from the first ALMA observations was that the formation heights are less clear than what was assumed. This development is expressed in the mentioned Chintzoglou et al. papers. It seems likely that the contribution functions can have multiple components. For instance, the ALMA Band 3 observations by Wedemeyer et al. (2020) show imprints of coronal loops which are clearly located well above the assumed formation height. Forward modelling of mm continuum emission sensitively depends on realistic electron densities, which on the other hand makes the comparison with ALMA observations a critical step in advancing these models and thus our understanding of the solar chromosphere. Strictly simultaneous observations over an extended frequency range, as expected from AtLAST, would be pivotal for better constraining the formation heights and contribution functions, thus enabling more reliable interpretation of the observations. We added a few sentences at the end of Sect. 2.1. ========================================================================

 (4) "Despite already large success of ALMA in advancing solar physics (e.g. Brajša et al., 2018; Heinzel et al., 2022; Labrosse et al., 2022; Menezes et al., 2022; Oliveira e Silva et al., 2022; Rodger et al., 2019; Selhorst et al., 2019; Shimojo et al., 2017a; Skokić et al., 2023; Valle Silva et al., 2021), there are important science cases that require different observational capabilities"

 I recommend that you split these advances into a few themes and at least mention what themes these are that ALMA has advanced. It's the second claim in the introduction of big contributions to advancements in the field with no specific examples yet provided to the reader. Further interesting advances from authors not associated with this work also perhaps warrant consideration in such a list. for example Multiple papers by Da Silva Santos, Chinzoglou, Molnar spring to mind, but there are also others [references 1-8]. I am not requesting an in-depth discussion or an exhaustive list, but some context for the reader who is not familiar with these works. 

 ANSWER> The aim was not to provide a review of ALMA results but we nonetheless added a few more references and modified the sentence.  ========================================================================

 (5 - Key Science Cases) "The chromosphere is an integral part of the solar atmosphere and as such plays an important role in the transport of energy and matter. These are essential for understanding the heating of the corona, the origins of the solar wind, and the drivers of solar activity and space weather. There is a plethora of physical processes involved, which are entangled in complicated dynamic ways, rendering the understanding of the workings of our Sun a challenging undertaking."

 (a) citations needed.

 (b) "The chromosphere is an integral part of the solar atmosphere and as such plays an important role in the transport of energy and matter."

 This sentence currently has low informational value. I suggest either

 - simply stating that the chromosphere plays an important role in the transport of energy and matter with some citations

 or

 - actually explaining what you mean by a little in terms of it being "an integral part" of the solar atmosphere and why. For example, one could argue this in terms of the behaviour changes either side of the plasma beta=1 layer, wave propagation, and non-equilibrium conditions. I am not a fan of characterising something as complicated or shrouded in mystery for the reader to understand that there are unresolved, interesting, and valuable science questions. It would be great if the authors could add some detail to pull back the curtain of mystery and complexity a little. 

 ANSWER> The sentences contained “low informational value” because they were only meant as an introduction for more detailed information given in that section. Also, this article is not meant to be a review article. However, we took the comment seriously and added extra information to address the challenges more specifically. Moreover, the moved the paragraph to the beginning of Sect. 2.1 as this has now become an introduction to the (thermal) structure of the chromosphere as a science case.  ========================================================================

 (6) "As detailed in the sections below, AtLAST (Mroczkowski et al., 2024) opens up a new window to science cases that cannot be addressed with existing sub-mm telescopes. In addition, some solar science cases for ALMA (see Bastian et al., 2018; Wedemeyer et al., 2016, and references therein; see also Figure 1) and LLAMA (Lépine et al., 2021) are potential solar use cases for AtLAST, too, thus creating synergies between these observatories. These science cases concern the thermal structure of the solar atmosphere including the thermal structure of prominences and filaments, the related transport of energy and mass, and phenomena related to solar activity. In the following, the key science cases for AtLAST are highlighted."

 (a) I do not see the authors linking Figure 1 to anything stated above. The images show two time points in the solar cycle. To clarify, I am certain that solar cycle data from ALMA has valuable information. However, since none is mentioned or explained here, figure 1 appears to be only a visually satisfying image. Please provide some context in the caption or the text. This could even be a reference to the later point in the paper where this figure is discussed. 

 ANSWER> At this point, the figure is only meant to give a first impression of how the Sun looks like at mm wavelengths. We changed the text to “ALMA (see Bastian et al., 2018; Wedemeyer et al., 2016, and references therein; see also Figure 1 for examples of ALMA single-dish scans of the Sun)”. ========================================================================

 (6b) Are you saying these listed science cases will be addressed in the following sections? If so, then I request you say that. If not then the statement is too brief to provide any information of value to the reader and I recommend adding citations, context, or both. 

 ANSWER> We do not understand the problem. The last sentence reads “In the following, the key science cases for AtLAST are highlighted.” and thus clearly indicates that detailed information is given in the following sections. We added the word “subsections” to make this more obvious.  ========================================================================

 (7a - Sect. 2.1) The first part of this section is very nicely stated, although again lacking any citations beyond one early paper relating to the coronal heating problem. Please add citations.

 ANSWER> We added more recent references.  ========================================================================

 (7b) "In principle, identifying these mechanisms and understanding their contributions requires quantitative and precise measurements that capture the thermal, magnetic, and kinetic state of chromospheric plasma over time and in three spatial dimensions. AtLAST has the potential to provide such data."

 At this point in the paper, the reader has not been provided with an overview of the capabilities of the instrument, nor have you provided citations with reasoned explanations or estimated of what observational capabilities could be required to derive these properties at the cadences and spectral/spatial resolutions required. Thus, unless the reader is already previously aware of all these details they have to accept the claim on trust. As stated in the summary I recommend this concern could be adequately addressed with context, references to later sections, or re-structuring of the paper. 

 ANSWER> This is clearly a misunderstanding that is understandable as the referee likely received the manuscript without the necessary context. This manuscript, together several others covering other astronomical topics, is a result of the first design phase. At this point, the telescope properties are defined but no instruments have been designed. The purpose of this article is to outline what AtLAST - based on the telescope specs - could do and how a corresponding instrument should be designed to make full of use of that. We see that a reader may indeed not understand this approach. We made therefore changes to clarify this point at the end of the introduction (Sect. 1).  ========================================================================

 (8 - Sect. 2.2 Solar Flares) "AtLAST is poised to make significant contributions in addressing numerous unresolved inquiries pertaining to solar flares, a prominent conundrum within contemporary solar physics that continues to be a highly dynamic area of investigation. Although numerous intricacies remain shrouded in mystery, it is evident that solar flares manifest as a result of the dynamic reconfiguration and interconnection of magnetic fields within the solar atmosphere."

 Citations needed here. But I thank the authors of this section for providing sufficient citations elsewhere. 

 ANSWER> We added a few more references.  ========================================================================

 (9) "And yet, it cannot be ruled out that even stronger flares exceeding the Carrington event of 1859 could occur with disastrous consequences for our technology-dependent modern society. Such so-called super-flares are observed for other (solar-like) stars (Maehara et al., 2012) (see Section 2.5). The study of strong solar flares, to which AtLAST can contribute in new ways, is thus of utmost importance."

 At this point in the paper without any solid evidence provided of how AtLAST can contribute, we are taking this "utmost importance" on faith. I recommend the authors to talk about the ways in which it can contribute explicitly. For example describing the observational capabilities, cadences, resolutions, atmospheric parameters, and how these relate to the flare science questions identified. 

 ANSWER> The “utmost importance" referred to studying flares and not flare observations with AtLAST. We rephrased and extended this part and now point at AtLAST capability to observe the whole disk of the Sun at high cadence, which increases the chance to capture the early stages of flares more systematically. This feat will indeed provide essential new data.  ========================================================================

 (10 - Figure 2)

 I assume the close association of mm-continuum radiation with brightness temperature will providing information to answer particular science questions. The relationship in flares is nicely demonstrated by the figure. Please point this out, and mention some of the science cases this will address and explain how the information will help. 

 ANSWER> The close relation between continuum flux and brightness temperature is mentioned already in the introduction as it is a fundamental reason for observing at mm wavelengths. The scientific implications are discussed in Sect. 2. For instance, this aspect is particularly important for Sect. 2.1. We revised Sect. 2 correspondingly.  ========================================================================

 (11) "Observations of such sub-mm flares might also provide key information about the formation of hot onsets in solar flares (da Silva et al., 2023; Hudson et al., 2021)."

 What sort of key information? Why "might"? Can you analyse whether the information obtained would help address the science questions? I recommend talking a bit about the science questions, the observational requirements and how AtLAST can provide observations that meet those requirements. 

 ANSWER> We updated this sentence but refrain from expanding this point too much as the article is not intended to become a complete review of all solar science at mm wavelengths.  ========================================================================

 (12) "Addressing these aspects with AtLAST would require high spectral, spatial and temporal resolution and preferably full polarisation"

 And can/do we have that? The observational capabilities of the instrumentation have not been summarised or discussed. Please address whether you believe this is feasible and justify your position to the reader. 

 ANSWER> As stated above, the intention of this manuscript is opposite. As written in that section, it would be important to probe the magnetic structure of the chromosphere, which would require polarisation capabilities. This requirement, which is met by the telescope, must then also be met by an instrument, which has not been designed yet. This manuscript provides a first impression of the instrumental requirements for making AtLAST able to address key solar science cases.  ========================================================================

 (13 - Solar Prominences) "These prominences are among the longest-lasting solar phenomena with lifetimes of several days to weeks. The large field of view of AtLAST, combined with its good spatial resolution and the ability to observe the Sun on daily bases for extended periods, will offer us opportunities to study the questions of the prominence origin, evolution, and eventual instability."

 The paragraph below this one makes a nice case for mm studies of prominences.

 I recommend that the discussion could be supplemented in several ways:

 (a) citations where appropriate.

 (b) are there physical processes relating to prominence formation/evolution/instability that occur at scales and cadences you anticipate to be evident at AtLAST's resolution (please also state that resolution), which are not currently accessible via existing solar mm instrumentation?

 (c) it may also be worth mentioning the validation and calibration you could perform on estimates of plasma quantities that are otherwise made using inferences from complicated optically thick radiative transfer. 

 ANSWER> We edited the section accordingly.  ========================================================================

 (14) "because the drivers that make the prominence magnetic field configurations unstable and erupt are still not well understood." & "However, it is unclear exactly what sets off these eruptions." Repetition

 ANSWER> In connection with replying to other referee comments, we edited this section and resolved the issue.  ========================================================================

 (15 - Solar Activity Cycle) I recommend that this section could do with a few more citations for the results you discuss - if possible not so dominantly from one of your authors, Menezes. Also please define TP in the text. 

 ANSWER> We appreciate the comment but would like to point out that there are not many studies monitoring the solar cycle at mm/radio wavelengths that could be cited here, which is exactly why AtLAST could contribute significantly to this science case. However, we added a several related references to this section.  ========================================================================

 (16) "Please note that the Sun’s rotation depends on latitude with a period of down to 24 days at the equator (see, e.g., Schou et al., 1998; Snodgrass, 1983). Any long-lived feature like an Active Region would thus move across the visible disk of the Sun in less than two weeks. However, at the resolution anticipated for AtLAST (see Figure 3), features like Active Regions and the sunspots (Solanki, 2003) therein would evolve significantly on a daily timescale. This use case would therefore be addressed best via full-disk mapping of the Sun at multiple frequency bands, once per day."

 Please clarify which use case you are referring to - Are you talking about studying the solar cycle in general or some specific observational case within that? 

 ANSWER> We clarified this point in the corresponding sentence.  ========================================================================

 (17) "New insights regarding the Sun’s activity variations and thus the long-term evolution of our host star could be transferred to other stars. This Sun-as-a-star approach has recently received increasing attention in the context of next-generation exoplanet observations for which it is crucial to separate observable exoplanet signatures from the host star’s “background radiation”."

 Citations needed. 

 ANSWER> We added some citations.  ========================================================================

 (18 - The Solar Stellar Connection) This section does several things very well that should be highlighted for interpreting my other comments in this review.

 (a) cites papers when claims regarding physical processes are made that relate to the science cases. (b) relates stated capabilities and resolutions/cadences of the instrument directly to the use cases under discussion.

 The spectrum of cited authors and findings within the field is rather limited, I suggest a more comprehensive coverage, but other than this the I have no notes on the section. 

 ANSWER> Thanks for this positive comment. We added more references, in particular regarding exo-space-weather.  ========================================================================

 (19 - Sect. 3) The information in this section would have been useful to know earlier, or at least be referenced from the earlier sections. Also given some of the abbreviations are defined in section 3 and used in section 2 I would assume there was already some debate regarding this ordering. Please make section 2 easier to follow for first time readers by linking the claims to information from section 3. 

 ANSWER> We follow the suggestion and added cross-references to Sect. 3 at appropriate places.  ========================================================================

 (20) "The Sun evolves on short time scales which prohibits long integration/scan times."

 I recommend changing the word "prohibits" to another word, as this doesn't really prohibit long integration times, it just devalues them for resolving transient structures. 

 ANSWER> We replaced “prohibits" with "requires correspondingly short". ========================================================================

 (21) "While it has yet to be seen if spectral lines, mostly hydrogen recombination lines and potentially CO, can be detected at millimeter wavelengths, they would have large diagnostic potential for assessing the thermal, kinetic and magnetic state of the chromospheric plasma."

 Could you comment on whether there is any ongoing effort to look into the existence of these lines within the proposed spectrum? Do you intend to conduct such a search in the early use cases? 

 ANSWER> That question is hard to answer at the current stage. It would ultimately depend on the instruments that would be built for AtLAST because the detection and then the use as diagnostics of these spectral lines would require sufficient spectral resolution. However, at this point it is unclear what these requirements are exactly and if/how an adequate instrument would be built. These questions should and will be address in the next development phase of AtLAST.   ========================================================================

 (22 - Sect. 3.1.3) Regarding the points made in the final paragraph, please could you comment on the outlook for being able to cover the necessary for studying flares? Perhaps some mention of this would be useful in the text too. I do believe there would be very useful information to glean about flare ribbons, condensation and evaporation even from a more limited dynamic range. ANSWER> Such information would certainly be useful but depends on the properties of the available instruments which are not defined at all at this stage. We believe that the paragraph adequately outlines the requirements and resulting challenge and that any more specific discussion of selected aspects of flares in this paragraph would be too speculative. Rather, these points are material for a more detailed paper in connection with an instrumental design study.  ========================================================================

 (23 - Sect. 3.1.4) "magnetograms obtained from the spectral lines in the photosphere (i.e., the Sun’s surface layer)"

 This is the 4th instance of the word photosphere. If you wish to explain what it is, I would recommend doing so at the first instance. 

 ANSWER> At first use, we write now “photosphere (i.e., the Sun’s surface layer)”. ========================================================================

 (24) "Reliably measuring the magnetic field in the solar chromosphere above, i.e. the layer that would be observed with AtLAST, is still a current technological challenge, again with much diagnostic potential."

 Citations to works from Pietrow, Morosin, and Vissers plus any the authors wish would be useful here [Refences 9-11]. 

 ANSWER> That is a good suggestion. We added the references.  ========================================================================

 (25) "Early observations with ALMA at a frequency of 100 GHz, which were obtained as science verification data in Band 3 (ADS/JAO.ALMA#2011.0.00011.E),"

 I recommend considering if this is the best way to cite this data. Perhaps providing a footnote or a reference to its description elsewhere in the document that says what this is and how to obtain it. 

 ANSWER> We do not agree and keep the ALMA project ID where it is. ALMA policy demands that all IDs of employed data are clearly specified.  ========================================================================

 (26 - Sect. 3.2.1) "with respect to the single-dish Total Power scans with ALMA antennas"

 ANSWER> TP is now unabbreviated. I agree with the final statement of this section, that would be a most welcome addition to our field. ========================================================================

 (27- Sect. 3.3 Instrumentation) "the resulting diameter of the FOV is approximated as d fov ≈ ..."

 For clarity, I recommend using a format that makes it explicitly clear what is, and what is not, under the large square root. This could also be achieved satisfactorily with parentheses.

 ANSWER> Done.  ========================================================================

 (28 - Sect. 3.3.1) "The details of the detector setup have to be investigated in detail. That includes the spacing between pixels, the total number of pixels, and the resulting instantaneously covered FOV. A fast small circular scanning pattern could be used to fill intermediate pointings. Combining data from circular scans (if possible on a second time scale or at least <10 s) would then allow to produce data with higher angular resolution. A detailed study is currently in preparation (Kirkaune et al. in prep.)."

 Have the matters been studied in detail, or are being studied in detail (in prep)? If the former, I would recommend an additional citation to a published work referenced in first part of this paragraph. 

 ANSWER> We write now “Potential scanning strategies are currently being investigated (Kirkaune et al. in prep.).” ========================================================================

 (29 - Sect. 3.3.2) "Such a detector would allow for exploiting the spectral domain but comes at the price of a much smaller number of detector elements as compared to multi-chroic continuum cameras (see Section 3.3.1)," Why is this? Simply by re-assignment of those pixels? and a typo later: overal -> overall 

 ANSWER> We write now “...only with a much smaller number of detector elements as compared to multi-chroic continuum cameras due to the higher cost per pixel…” ========================================================================

 (30 - Sect. 3.3.3) "In any case, a realistic evaluation of the scientific potential of IFUs will require a dedicated study." Please comment on whether you have plans to perform that study. 

 ANSWER> This is simply a statement with no indication who will do it when. One of the next steps (in a few years) will be the initial design phase of potential instruments for AtLAST. It is too early to say anything more specific right now.

# Reviewer report: Reviewer response for version 1

**DOI:** 10.21956/openreseurope.18862.r42516

**Published:** 2024-08-08

**Authors:** Francesco Pecora

**Affiliations:** 1 University of Delaware, Newark, Delaware, USA

Wedemeyer et al. presented a white paper on the technical properties of the AtLAST observatory that are needed to advance the current and planned generations of observatories. They also discuss a series of science questions that could be addressed with AtLAST including the atmospheric heating problem and the investigation of solar flares.

 Below are some comments that would hopefully improve its clarity.

1. Abstract. Typo, “simultaneously” should be “simultaneous”.
2. Abstract. In the last two paragraphs, if I understand correctly, it is suggested that AtLAST could observe other stars. However, in the science cases, this opportunity is not addressed. Why?
3. Section 2. If authors think it is appropriate, they could describe the potential synergy with the upcoming PUNCH mission (DeForest et al. 2022 10.1109/AERO53065.2022.9843340). For instance, it could be interesting to have AtLAST detailed observations at the sun’s surface and match them with unprecedented observations of CMEs.
4. Section 2.1 end of first paragraph. It is probably advisable to define “late-type stars”.
5. Section 2.1. The phenomena candidate for heating processes may include turbulence. Providing some references could be appropriate too, e.g. Cranmer (2002 [Ref - 2]) (https://doi.org/10.1023/A:1020840004535) for a review.
6. Section 2.2 end of second paragraph (“And yet...”). It is potentially interesting to add a point of discussion based on Gosling (1993 [Ref - 3]) ( https://doi.org/10.1029/93JA01896)
7. Section 2.4 third paragraph (“Please note…”), the authors could mention the rotation period close to the poles as well.
8. Section 3.1, point (ii) is slightly unclear. Did the authors intend that to observe fast phenomena, high temporal resolution is needed? On the same paragraph, what do the authors mean with “large dynamic range”?
9. Section 3.1.1. “With an aperture…” the authors point to Fig.4 that has units of GHz. For ease of the reader, would it be possible to either express relevant quantities in GHz in this paragraph or add another axis that makes direct contact with the units of this paragraph?
10. Sec. 3.1.1 second paragraph. “Scanning…longer”. Can it be quantified how long?
11. Fig. 4 caption. Why are the frequencies of 90GHz and 660GHz relevant? In the text it seems that 100GHz and 950GHz are mentioned.
12. Sec. 3.1.1 last paragraph. Define formation height range.
13. Sec. 3.1.4 last paragraph. When is Cycle 10?
14. Sec. 3.2 “10 min long…”. How much of the sun’s surface would this cover?
15. Same paragraph, define ToO.
16. The three methods described in Sec.s 3.2.1-3.2.3 are interesting. Is it possible to explicitly state what would be the spatial resolution, full FOV and temporal cadence for each one of them?

Is the case presented with sufficient detail to be useful for teaching or other practitioners?

Yes

Is the work clearly and accurately presented and does it cite the current literature?

Yes

If applicable, is the statistical analysis and its interpretation appropriate?

Not applicable

Are all the source data underlying the results available to ensure full reproducibility?

Not applicable

Are the conclusions drawn adequately supported by the results?

Yes

Is the background of the case’s history and progression described in sufficient detail?

Yes

Reviewer Expertise:

Turbulence

I confirm that I have read this submission and believe that I have an appropriate level of expertise to confirm that it is of an acceptable scientific standard.

## References

[1] : Polarimeter to UNify the Corona and Heliosphere (PUNCH): Science, Status, and Path to Flight. *2022 IEEE Aerospace Conference (AERO)* .2022;1-11 Reference source

[2] : *Space Science Reviews* .2002;101(3/4) : 10.1023/A:1020840004535 229-294 10.1023/A:1020840004535

[3] : The solar flare myth. *Journal of Geophysical Research: Space Physics* .1993;98(A11) : 10.1029/93JA01896 18937-18949 10.1029/93JA01896

# Response

**Published:** 2024-11-13

**Authors:** Sven Wedemeyer

We would like to thank the referee for the useful suggestions. We considered all comments, which resulted in an improved manuscript. Please note that the revised version takes into account the comments from all three referees. We added 81 additional references and updated Figs. 3 and 4. In the following, we address all comments in detail.  ========================================================================

 1. Abstract. Typo, “simultaneously” should be “simultaneous”. ANSWER> ok. ========================================================================

 2. Abstract. In the last two paragraphs, if I understand correctly, it is suggested that AtLAST could observe other stars. However, in the science cases, this opportunity is not addressed. Why? ANSWER> That is mostly due to the initial topics covered in the first AtLAST design study (which resulted in several manuscripts like this one). Those topics did not include stellar observations. Given the synergies between solar and stellar physics, we wanted to at least point at this connection. The manuscript therefore (already in its submitted form) contains a section on the solar-stellar connection (Sect. 2.5).  ========================================================================

 3. Section 2. If authors think it is appropriate, they could describe the potential synergy with the upcoming PUNCH mission (DeForest et al. 2022 10.1109/AERO53065.2022.9843340). For instance, it could be interesting to have AtLAST detailed observations at the sun’s surface and match them with unprecedented observations of CMEs. ANSWER> As far as we can see, the PUNCH mission will be launched in about 4 months with nominal mission duration of 2 years. This mission will thus complete in 2026 (plus any extension) and thus well before AtLAST would commence operations. It seem therefore strange to mention PUNCH in connection with AtLAST.  ========================================================================

 4. Section 2.1 end of first paragraph. It is probably advisable to define “late-type stars”. ANSWER> We write now instead "for all stars with notable surface convection, i.e. in particular main sequence stars of spectral types G-M." ========================================================================

 5. Section 2.1. The phenomena candidate for heating processes may include turbulence. Providing some references could be appropriate too, e.g. Cranmer (2002 [Ref - 2]) (https://doi.org/10.1023/A:1020840004535) for a review. ANSWER> While it is certainly correct that turbulence should be mentioned in this context, the same is true for a large range of other processes and effects. We do not intend to give a complete review on this topic but expanded this part with two sentences and several references.  ========================================================================

 6. Section 2.2 end of second paragraph (“And yet...”). It is potentially interesting to add a point of discussion based on Gosling (1993 [Ref - 3]) (https://doi.org/10.1029/93JA01896) ANSWER> We disagree with the referee. The claim put forward by Gosling in 1993 was controverse from the beginning and been disproven countless times with increasing support from new and more detailed observations. Hugh Hudson (among the authors of this article) summarised it as follows in the paper by Hudson et al. (1993): “In a recent paper Gosling (1993) claims that solar flares are relatively unimportant for understanding the terrestrial consequences of solar activity, and argues that coronal mass ejections (CMEs) produce the most powerful terrestrial disturbances. This opinion conflicts with observation, as it is well known that CMEs and flares are closely associated, and we disagree with Gosling's insistence on a simplistic cause-and-effect description of the interrelated phenomena of a solar flare. In this brief response we present new Yohkoh data and review older results that demonstrate the close relationships among CMEs, flares, filament eruptions, and other forms of energy release such as particle acceleration.” The progress in our understanding of the origins and impact of space weather since 1993 is substantial, providing solid and  convincing arguments against the (simplistic) claim by Gosling.  ========================================================================

 7. Section 2.4 third paragraph (“Please note…”), the authors could mention the rotation period close to the poles as well. ANSWER> Done.  ========================================================================

 8. Section 3.1, point (ii) is slightly unclear. Did the authors intend that to observe fast phenomena, high temporal resolution is needed? On the same paragraph, what do the authors mean with “large dynamic range”? ANSWER> Sorry but we do not understand why this point is not clear. As stated, the “Sun evolves on short time scales which requires correspondingly short integration/scan times”. Consequently, any observation of small details requires fast imaging. Regarding the dynamic range, we added in parentheses “(i.e., a high ratio between the maximum and minimum measurable brightness temperatures)”.  ========================================================================

 9. Section 3.1.1. “With an aperture…” the authors point to Fig.4 that has units of GHz. For ease of the reader, would it be possible to either express relevant quantities in GHz in this paragraph or add another axis that makes direct contact with the units of this paragraph? ANSWER> Fair point. We now mention both wavelengths and frequencies.  ========================================================================

 10. Sec. 3.1.1 second paragraph. “Scanning…longer”. Can it be quantified how long? ANSWER> It is difficult to give a definite answer but simply based on the telescope diameters we now write “longer (on the order of (50m/12m)2≈17 times, i.e. ~3 hours)”  ========================================================================

 11. Fig. 4 caption. Why are the frequencies of 90GHz and 660GHz relevant? In the text it seems that 100GHz and 950GHz are mentioned. ANSWER> We added the following to the caption: “which we here define as a minimum requirement for the frequency range.” While we happily would use an instrument with a frequency range up to 950 GHz, the high frequency come with an accordingly small beam, thus making scanning the full disk at reasonable cadence challenging while the observing conditions are typically much more demanding for the highest frequencies. It thus not clear if the additional technical challenges are worth the resulting data for the highest frequencies - except for some lucky cases which of course could be scientifically interesting for individual targets but not so much for long series of daily maps. See Sect. 3.1.2. ========================================================================

 12. Sec. 3.1.1 last paragraph. Define formation height range.

 ANSWER> We refer now to Sect. 2.1 in which the formation height aspect is now introduced. ========================================================================

 13. Sec. 3.1.4 last paragraph. When is Cycle 10?

 ANSWER> Done.  ========================================================================

 14. Sec. 3.2 “10 min long…”. How much of the sun’s surface would this cover?

 ANSWER> Also full-disk maps. Now clarified in the manuscript.  ========================================================================

 15. Same paragraph, define ToO.

 ANSWER> Done.  ========================================================================

 16. The three methods described in Sec.s 3.2.1-3.2.3 are interesting. Is it possible to explicitly state what would be the spatial resolution, full FOV and temporal cadence for each one of them? ANSWER> Sorry but no such details can be given at this points. These numbers will depend on the instrumental design which itself may well depend on technological aspects that are currently under development and therefore hard to pin down. This manuscript attempts to give a first (qualitative) impression of solar observations with AtLAST and why it is worthwhile to invest more effort into developing a suitable instrument.

# Reviewer report: Reviewer response for version 1

**DOI:** 10.21956/openreseurope.18862.r42510

**Published:** 2024-08-05

**Authors:** Alexander G.M. Pietrow

**Affiliations:** 1 Leibniz Institute for Astrophysics Potsdam, Potsdam, Germany

This manuscript discusses the construction and utilization of the ⌀ 50-meter single-dish Atacama Large Aperture Submillimeter Telescope (AtLAST). The primary goal is to provide a comprehensive sub-mm diagnostic tool for studying the Sun’s chromosphere, and that of other stars. This telescope will help fill the resolution gap around zero in Fourier space, and its large size will allow for overlap between the ALMA resolution space in a way that the existing 12-meter single dishes cannot. This is a timely and valuable project that will help further explore solar and stellar spectra beyond the infrared. 

 However, several aspects of the current version of the paper are unclear. Therefore, I suggest making modifications to address these issues before the paper is accepted for indexing. Additionally, some minor points could be improved in the revised manuscript. I have both comment types below in order of appearance. All comment locations are based on the PDF of the first version of the manuscript. 

1. **Page 5, second column, second paragraph, first half**; This paragraph gives a good overview of solar physics done in sub-mm wavelengths, but does not emphasize the importance of these bands in combination with other wavelength bands. Especially for spectroscopic inversions and differential emission measures. (e.g. [Ref 1-6]) Could the authors mention these aspects and cite relevant literature? 
2. **Page 5, second column, second paragraph, second half**; I believe that this is the location where AtLASTs capabilities (proposed wavelength bands, cadences, resolution, etc.) should be summarised for the reader. This currently happens in multiple locations across the manuscript and an overview would be very helpful.  Table1 from  [Ref - 7] is a good example. 
3. **Page 6, second column, last paragraph**; This paragraph should be more quantitative with examples and citations. 
4. **Page 7, first column, second paragraph**; The Carrington event has an energy of the same order of magnitude as the 1e32 erg described in the same paragraph ( ~5e31 erg, e.g. [Ref - 8]) How much stronger (in orders of magnitude) are solar flares expected to be? [Ref - 9] puts the limit at ~1e36 for flares in general.
5. **Page 7, second column, second paragraph**; I believe that the word associated should be correlated instead.
6. **Page 7, figure 2**; Please mark the AtLAST bands on the figure, or mention them in the caption. 
7. **Page 8, section 2.3**; Perhaps a link could be made with prominence models and how measurements in the sub-mm would provide valuable constraints for such models (e.g. [Ref 10-12]).
8. **Page 9, first column, first paragraph**; The acronym TP has not been defined.
9. **Page 9, first column, second paragraph**; It is not fully clear if this paragraph suggests a high-cadence observing mode, or daily full disk images. I believe that it is the latter, but in that case, solar flares should be removed from this list as the odds of capturing them are very low for daily maps. Unless the authors mean that tracking the evolution of active regions will shed light on flare physics, in which case this should be clarified.
  Additionally, the Solanki reference should be moved to the first mention of sunspots and perhaps [Ref - 13] could be added where it is pointed out that active regions move at different velocities than the quiet Sun. 
10. **Page 9, second column, second paragraph**; I believe that a bigger summary should be given concerning sun-as-a-star observations in other wavelengths and how AtLAST would add to these programs. Some suggested citations on instruments, and Sun-as-a-star work done with these instruments concerning Sun-as-a-star flares can be found in [Ref 14-19]. A short discussion of how resolved vs unresolved flare studies [Ref 17-19] help with understanding flare observations would fit here. Additionally, if a flare is caught at a high cadence with a limited FOV, would it be feasible to turn this into a sun-as-a-star observation using a method similar to e.g. [Ref - 20]? 
11. **Page 9, figure 3**; Please add an additional panel that shows the resolution with an ALMA SD dish. This would give a good comparison between existing and proposed instrumentation. 
12. **Page 10, first column one, third paragraph**; “...still no confident detection of a stellar coronal mass ejection…” This is a very strong statement that would need to discuss proposed detections and explain why they are not confident. I suggest saying that only a relatively low number of CMEs has been detected so far, and cite [Ref 21-29].  
13. **Page 10, first column one, fourth paragraph**; “...very short integration times and…” Could you give an order of magnitude value here? 
14. **Page 10, second column one, second paragraph**; The last part of this paragraph should be moved to the front (See point 2).
15. **Page 10, second column one, second paragraph**; “On-the-fly scanning” How is this different from taking a mosaic? If it is not, perhaps it could be mentioned that this is similar or equivalent to taking a mosaic. In section 3.2.3 (pp13) they are equated to mosaics.
16. **Page 11, figure 4**; Please add a wavelength axis to panels a and b, as was done in Fig. 2.
  Additionally, should the vertical solid line in panel b also be dashed? 
17. **Page 12, first column, second paragraph**; The cited source does not discuss cold temperatures below ~4500 K, perhaps the authors could add a small discussion on these lower temperatures and their implications, given that they are below that of the temperature minimum. Citation 4 (last part of section 4.1) offers a good summary and an explanation of why other chromospheric proxies are not sensitive to these temperatures. 
18. **Page 12, first column, second paragraph**; “...the 1.5m-aperture Solar Submillimeter Telescope (SST)..” This telescope has already been introduced in the first paragraph of section 2.4, although without the citation. 
19. **Page 12, first column, second paragraph**; Perhaps a figure could be added of the FAL-C model (temperature vs height) and the approximate heights at which the telescope different bands are sensitive be marked.  
20. **Page 13, first column, first paragraph**; ToO -> undefined acronym 
21. **Page 13, first column, second paragraph**; “..would require long scan times.” Please give an order of magnitude estimate. The latter part of this paragraph does explain it somewhat, but I suggest this is edited to be more clear. 
22. **Page 13, first column, third paragraph**; “..high cadence.” -> please give an estimate.
23. **Page 13, second column, second paragraph**; “...slightly different science cases…” Please specify. 
24. **Page 13, second column, second paragraph**; equation on the last line. Please give in larger mode for readability and give a numerical approximation of the constant. 
25. **Throughout**; The manuscript has a preference for frequency over wavelength when discussing bands, however, it gives both at some locations in the text. I suggest that this is homogenized and that the wavelength is also given to improve readability for those outside of radio astronomy.

Is the case presented with sufficient detail to be useful for teaching or other practitioners?

Partly

Is the work clearly and accurately presented and does it cite the current literature?

Partly

If applicable, is the statistical analysis and its interpretation appropriate?

Not applicable

Are all the source data underlying the results available to ensure full reproducibility?

Not applicable

Are the conclusions drawn adequately supported by the results?

Yes

Is the background of the case’s history and progression described in sufficient detail?

Yes

Reviewer Expertise:

Solar physics, Sun-as-a-star observations, flares, and spectropolarimetric inversions.

I confirm that I have read this submission and believe that I have an appropriate level of expertise to confirm that it is of an acceptable scientific standard, however I have significant reservations, as outlined above.

## References

[1] : Temperature constraints from inversions of synthetic solar optical, UV, and radio spectra. *Astronomy & Astrophysics* .2018;620: 10.1051/0004-6361/201833664 10.1051/0004-6361/201833664

[2] : The multi-thermal chromosphere. *Astronomy & Astrophysics* .2020;634: 10.1051/0004-6361/201937117 10.1051/0004-6361/201937117

[3] : ALMA observations of transient heating in a solar active region. *Astronomy & Astrophysics* .2020;643: 10.1051/0004-6361/202038755 10.1051/0004-6361/202038755

[4] : Evaluating Non-LTE Spectral Inversions with ALMA and IBIS. *The Astrophysical Journal* .2022;933(2) : 10.3847/1538-4357/ac6f00 10.3847/1538-4357/ac6f00

[5] : An ALMA Observation of Time Variations in Chromospheric Temperature of a Solar Plage Region. *Frontiers in Astronomy and Space Sciences* .2022;9: 10.3389/fspas.2022.908249 10.3389/fspas.2022.908249

[6] : First looks at solar active regions with ALMA. *Frontiers in Astronomy and Space Sciences* .2022;9: 10.3389/fspas.2022.1025368 10.3389/fspas.2022.1025368

[7] : Solar Observations with ALMA: A New Frontier. *Proceedings of the International Astronomical Union* .2022;18(S372) : 10.1017/S1743921322004586 28-37 10.1017/S1743921322004586

[8] : The 1859 space weather event revisited: limits of extreme activity. *Journal of Space Weather and Space Climate* .2013;3: 10.1051/swsc/2013053 10.1051/swsc/2013053

[9] : Statistical Analysis of Stellar Flares from the First Three Years of TESS Observations. *The Astrophysical Journal* .2022;935(2) : 10.3847/1538-4357/ac8352 10.3847/1538-4357/ac8352

[10] : 1.5D non-LTE spectral synthesis of a 3D filament and prominence simulation. *Astronomy & Astrophysics* .2023;670: 10.1051/0004-6361/202244868 10.1051/0004-6361/202244868

[11] : Rotational Flows in Solar Coronal Flux Rope Cavities. *The Astrophysical Journal Letters* .2023;953(1) : 10.3847/2041-8213/acea78 10.3847/2041-8213/acea78

[12] : MHD Simulation of Prominence-Cavity System. *Frontiers in Astronomy and Space Sciences* .2019;6: 10.3389/fspas.2019.00027 10.3389/fspas.2019.00027

[13] : Differential rotation of sunspot groups in the period from 1874 through 1976 and changes of the rotation velocity within the solar cycle. *Astronomy and Astrophysics* .1986;155(1) :87-98 Reference source

[14] : HARPS-N OBSERVES THE SUN AS A STAR. *The Astrophysical Journal* .2015;814(2) : 10.1088/2041-8205/814/2/L21 10.1088/2041-8205/814/2/L21

[15] : PEPSI deep spectra. *Astronomy & Astrophysics* .2018;612: 10.1051/0004-6361/201731631 10.1051/0004-6361/201731631

[16] : The Extreme Stellar-signals Project. III. Combining Solar Data from HARPS, HARPS-N, EXPRES, and NEID. *The Astronomical Journal* .2023;166(4) : 10.3847/1538-3881/acf83e 10.3847/1538-3881/acf83e

[17] : Sun-as-a-star Analyses of Various Solar Active Events Using Hα Spectral Images Taken by SMART/SDDI. *The Astrophysical Journal* .2022;939(2) : 10.3847/1538-4357/ac9730 10.3847/1538-4357/ac9730

[18] : A comparative study of two X2.2 and X9.3 solar flares observed with HARPS-N. Reconciling Sun-as-a-star spectroscopy and high-spatial resolution solar observations in the context of the solar-stellar connection. *Astronomy & Astrophysics* .2023; 10.1051/0004-6361/202347895 10.1051/0004-6361/202347895

[19] : Sun-as-a-star Study of an X-class Solar Flare with Spectroscopic Observations of CHASE. *The Astrophysical Journal* .2024;966(1) : 10.3847/1538-4357/ad3446 10.3847/1538-4357/ad3446

[20] : Center-to-limb variation of spectral lines and their effect on full-disk observations.2003; Reference source

[21] : Dynamics of flares on late-type dMe stars. I. Flare mass ejections and stellar evolution. *Astronomy and Astrophysics* .1990;238:249-249 Reference source

[22] : Dynamics of flares on late type dMe stars. II. Mass motions and prominence oscillations during a flare on AD Leonis. *Astronomy and Astrophysics* .1993;274:245-264 Reference source

[23] : High-velocity evaporation during a flare on AT Microscopii. *Astronomy and Astrophysics* .1994;285:489-496 Reference source

[24] : Spectrophotometry of flares and short time scale variations in weak line, and classical T Tauri stars in Chamaeleon. *Astronomy and Astrophysics* .1997;321:803-810 Reference source

[25] : Detection and high-resolution spectroscopy of a huge flare on the old M 9 dwarf DENIS 104814.7-395606.1. *Astronomy & Astrophysics* .2004;420(3) : 10.1051/0004-6361:20035644 1079-1085 10.1051/0004-6361:20035644

[26] : Investigating magnetic activity in very stable stellar magnetic fields. *Astronomy & Astrophysics* .2016;590: 10.1051/0004-6361/201527925 10.1051/0004-6361/201527925

[27] : A stellar flare−coronal mass ejection event revealed by X-ray plasma motions. *Nature Astronomy* .2019;3(8) : 10.1038/s41550-019-0781-4 742-748 10.1038/s41550-019-0781-4

[28] : Probable detection of an eruptive filament from a superflare on a solar-type star. *Nature Astronomy* .2021;6(2) : 10.1038/s41550-021-01532-8 241-248 10.1038/s41550-021-01532-8

[29] : Indications of stellar coronal mass ejections through coronal dimmings. *Nature Astronomy* .2021;5(7) : 10.1038/s41550-021-01345-9 697-706 10.1038/s41550-021-01345-9

# Response

**Published:** 2024-11-13

**Authors:** Sven Wedemeyer

We would like to thank the referee for the useful suggestions. We considered all comments, which resulted in an improved manuscript. Please note that the revised version takes into account the comments from all three referees. We added 81 additional references and updated Figs. 3 and 4. In the following, we address all comments in detail.  ========================================================================

 1. Page 5, second column, second paragraph, first half; This paragraph gives a good overview of solar physics done in sub-mm wavelengths, but does not emphasize the importance of these bands in combination with other wavelength bands. Especially for spectroscopic inversions and differential emission measures. (e.g. [Ref 1-6]) Could the authors mention these aspects and cite relevant literature? 

**ANSWER>** We edited the section accordingly.  ========================================================================

 2. Page 5, second column, second paragraph, second half; I believe that this is the location where AtLASTs capabilities (proposed wavelength bands, cadences, resolution, etc.) should be summarised for the reader. This currently happens in multiple locations across the manuscript and an overview would be very helpful.  Table1 from  [Ref - 7] is a good example. **ANSWER>** This is clearly a misunderstanding that is understandable as the referee likely received the manuscript without the necessary context. This manuscript, together several others covering other astronomical topics, is a result of the first design phase. At this point, the telescope properties are defined but no instruments have been designed. The purpose of this article is to outline what AtLAST - based on the telescope specs - could do and how a corresponding instrument should be designed to make full of use of that. We see that a reader may indeed not understand this approach. We made therefore changes to clarify this point at the end of the introduction (Sect. 1). A table as suggested (Ref -7) is not possible at this stage, simply because that information does not exist. 

 ========================================================================

 3. Page 6, second column, last paragraph; This paragraph should be more quantitative with examples and citations. 

**ANSWER>** While we appreciate the suggestion, we would like to emphasise that this article is not meant to be a complete review article. We considered all comments of all three referees already to an extent that expanded the manuscript over its intended scope. As mentioned earlier in the article, a more complete review over solar mm science cases is provided by, e.g., Wedemeyer et al. 2016. We mentioned that now at the beginning of Sect. 2.2. ========================================================================

 4. Page 7, first column, second paragraph; The Carrington event has an energy of the same order of magnitude as the 1e32 erg described in the same paragraph ( ~5e31 erg, e.g. [Ref - 8]) How much stronger (in orders of magnitude) are solar flares expected to be? [Ref - 9] puts the limit at ~1e36 for flares in general.

**ANSWER>** Thanks for this interesting addition. We added a corresponding sentence (but state the values provided by the given references).  ========================================================================

 5. Page 7, second column, second paragraph; I believe that the word associated should be correlated instead.

**ANSWER>** Changed.  ========================================================================

 6. Page 7, figure 2; Please mark the AtLAST bands on the figure, or mention them in the caption. 

**ANSWER>** Figure 2 is on page 5 in the submitted double-column version. Assuming that is the figure in question, we added a sentence to the caption.  ========================================================================

 7. Page 8, section 2.3; Perhaps a link could be made with prominence models and how measurements in the sub-mm would provide valuable constraints for such models (e.g. [Ref 10-12]).

**ANSWER>** We completely agree with the referee that the links between the prominence models and the sub-mm observations are important and interesting to describe. However, we would rather avoid an inclusion of a few superficial ideas about how the new observations will impact this rich field of solar physics. There are many sub-fields of the prominence modelling, for example the radiative transfer modelling, magnetic field simulations and extrapolations, 2.5D and 3D MHD models, and the latest complex models bringing many of these ingredients together. We could hardly do justice to the entire field in a few sentences, but we cannot review the state-of-the-art of the field in this paper either. However, we updated the section (also in response to the other referees’ comments).  ========================================================================

 8. Page 9, first column, first paragraph; The acronym TP has not been defined.

**ANSWER>** Done.  ========================================================================

 9. Page 9, first column, second paragraph; It is not fully clear if this paragraph suggests a high-cadence observing mode, or daily full disk images. I believe that it is the latter, but in that case, solar flares should be removed from this list as the odds of capturing them are very low for daily maps. Unless the authors mean that tracking the evolution of active regions will shed light on flare physics, in which case this should be clarified.

 Additionally, the Solanki reference should be moved to the first mention of sunspots and perhaps [Ref - 13] could be added where it is pointed out that active regions move at different velocities than the quiet Sun. 

**ANSWER>** Fair point. We added another sentence to clarify this and added the references as suggested.  ========================================================================

 10. Page 9, second column, second paragraph; I believe that a bigger summary should be given concerning sun-as-a-star observations in other wavelengths and how AtLAST would add to these programs. Some suggested citations on instruments, and Sun-as-a-star work done with these instruments concerning Sun-as-a-star flares can be found in [Ref 14-19]. A short discussion of how resolved vs unresolved flare studies [Ref 17-19] help with understanding flare observations would fit here. Additionally, if a flare is caught at a high cadence with a limited FOV, would it be feasible to turn this into a sun-as-a-star observation using a method similar to e.g. [Ref - 20]? 

**ANSWER>** We would not like to add a detailed discussion of resolved vs unresolved flare studies as the manuscript is not intended to be a comprehensive review. We have nevertheless extended the section accordingly and added the suggested references.  ========================================================================

 11. Page 9, figure 3; Please add an additional panel that shows the resolution with an ALMA SD dish. This would give a good comparison between existing and proposed instrumentation. **ANSWER>** We updated the figure accordingly.  ========================================================================

 12. Page 10, first column one, third paragraph; “...still no confident detection of a stellar coronal mass ejection…” This is a very strong statement that would need to discuss proposed detections and explain why they are not confident. I suggest saying that only a relatively low number of CMEs has been detected so far, and cite [Ref 21-29].  

**ANSWER>** We have toned down the statement and added all suggested references.  ========================================================================

 13. Page 10, first column one, fourth paragraph; “...very short integration times and…” Could you give an order of magnitude value here? 

**ANSWER>** Done.  ========================================================================

 14. Page 10, second column one, second paragraph; The last part of this paragraph should be moved to the front (See point 2).

**ANSWER>** See our answer to point 2. Instead we moved the paragraph addressing the chromosphere at the beginning of Sect. 2 into Sect. 2.1 and added two sentences with the basic telescope properties and refer to Sect. 3 for the definition of instrumental requirements. It is important to note that defining these requirements based on the basic telescope properties and the possible science cases is the main purpose of this manuscript, which can then serve as basis for future instrument design studies.  ========================================================================

 15. Page 10, second column one, second paragraph; “On-the-fly scanning” How is this different from taking a mosaic? If it is not, perhaps it could be mentioned that this is similar or equivalent to taking a mosaic. In section 3.2.3 (pp13) they are equated to mosaics. **ANSWER>** This is a misunderstanding. On-the-fly scanning is not the same as mosaics. We clarified this in Sect. 3.2.3. and added another sentence in Sect. 3.1.1. to explain the difference.  ========================================================================

 16.  Page 11, figure 4; Please add a wavelength axis to panels a and b, as was done in Fig. 2.

 Additionally, should the vertical solid line in panel b also be dashed? 

**ANSWER>** We updated the figure accordingly. ========================================================================

 17. Page 12, first column, second paragraph; The cited source does not discuss cold temperatures below ~4500 K, perhaps the authors could add a small discussion on these lower temperatures and their implications, given that they are below that of the temperature minimum. Citation 4 (last part of section 4.1) offers a good summary and an explanation of why other chromospheric proxies are not sensitive to these temperatures. 

**ANSWER>** While we appreciate the suggestion, the purpose of this section is to motivate the need for a high dynamic range. A discussion of low temperatures and the related trouble of applying a correct offset for interferometric observations with ALMA would divert too much from this purpose. We nonetheless expanded the text somewhat and added more references.  ========================================================================

 18.  Page 12, first column, second paragraph; “...the 1.5m-aperture Solar Submillimeter Telescope (SST)..” This telescope has already been introduced in the first paragraph of section 2.4, although without the citation. 

**ANSWER>** Fixed.  ========================================================================

 19. Page 12, first column, second paragraph; Perhaps a figure could be added of the FAL-C model (temperature vs height) and the approximate heights at which the telescope different bands are sensitive be marked.  

**ANSWER>** We would rather not want to use the FAL-C model as a reference. As we now describe in more detail in Sect. 2.1, i) the chromosphere is highly dynamic and that is not well represented by an average stratification as in the FAL-C model and ii) the exact formation heights of the mm continuum are currently an open question, which would make any such information uncertain. Instead, the manuscript makes it clear that AtLAST (like ALMA) would observe the chromosphere without going into too much detail.  ========================================================================

 20. Page 13, first column, first paragraph; ToO -> undefined acronym 

**ANSWER>** Fixed..  ========================================================================

 21. Page 13, first column, second paragraph; “..would require long scan times.” Please give an order of magnitude estimate. The latter part of this paragraph does explain it somewhat, but I suggest this is edited to be more clear. 

**ANSWER>** We refer now to the updated Sect. 3.1.1 where the longer scan times are now discussed  ========================================================================

 22. Page 13, first column, third paragraph; “..high cadence.” -> please give an estimate. **ANSWER>** Done. We also added why this is desirable as demonstrated by ALMA. ========================================================================

 23. Page 13, second column, second paragraph; “...slightly different science cases…” Please specify. 

**ANSWER>** We expanded this part.  ========================================================================

 24. Page 13, second column, second paragraph; equation on the last line. Please give in larger mode for readability and give a numerical approximation of the constant. 

**ANSWER>** We assume that this comment addresses the equation for the FOV. Please note that the typesetting was done by ORE based on our Latex (Overleaf) generated version. We hope that the editors can take up this suggestion when typesetting the final version.  ========================================================================

 25. Throughout; The manuscript has a preference for frequency over wavelength when discussing bands, however, it gives both at some locations in the text. I suggest that this is homogenized and that the wavelength is also given to improve readability for those outside of radio astronomy.

**ANSWER>** While it is more common to use frequency and while the conversion is relatively easy, we added both at several places to make the text easier to understand for a broader range of readers.
